# A Quasi-Experimental Controlled Study to Evaluate the Effects of a Kinesiologic Approach—The Canali Postural Method—To Posture Reprogramming for Non-Specific Low Back Pain

**DOI:** 10.3390/healthcare13080869

**Published:** 2025-04-10

**Authors:** Saverio Sabina, Daria Monteleone, Pierpaolo Mincarone, Patrizia Maiorano, Roberto Guarino, Maria Rosaria Tumolo, Carlo Giacomo Leo, Antonio Giordano, Mirko Zisi

**Affiliations:** 1 Institute of Clinical Physiology, National Research Council, Branch of Lecce, 73100 Lecce, Italy; saverio.sabina@cnr.it (S.S.); roberto.guarino@cnr.it (R.G.); mariarosaria.tumolo@unisalento.it (M.R.T.); carlogiacomo.leo@cnr.it (C.G.L.); 2 MOVE-mentis S.R.L., 47522 Cesena, Italy; 3 Sbarro Health Research Organization Italia, 10060 Candiolo, TO, Italy; monteleone.daria@gmail.com (D.M.); patriziamaiorano@gmail.com (P.M.); 4 Health Posture Training Lab, 80128 Naples, Italy; 5 Institute for Research on Population and Social Policies, National Research Council, Research Unit of Brindisi, 72100 Brindisi, Italy; 6 Department of Biological & Environmental Sciences & Technology, University of Salento, 73100 Lecce, Italy; 7 Sbarro Institute for Cancer Research and Molecular Medicine and Center for Biotechnology, College of Science and Technology, Temple University, Philadelphia, PA 19122, USA; president@shro.org; 8 Department of Medical Biotechnologies, University of Siena, 53100 Siena, Italy; 9 Habitus et Motus S.R.L., 40046 Alto Reno Terme, BO, Italy; mirko.zisifitness@gmail.com

**Keywords:** individualized structured exercises, musculoskeletal conditions, physical activity, public health, well-being

## Abstract

Background/Objectives: Low back pain (LBP) is the leading cause of disability worldwide, with most cases classified as non-specific low back pain (NSLBP). Various treatments exist, among which are physical exercises that promote flexibility, mobility and core stabilization, improving muscle function and body posture. The Canali Postural Method (CPM) is a kinesiological method that offers a personalized approach to postural reprogramming. This study compares the effects of the CPM and generic exercises in individuals with NSLBP. Methods: Subjects with NSLBP were engaged in a four-week intervention either based on a CPM reprogramming phase (CPM group) or generic exercises for the control group (CG). The CPM group underwent an assessment phase to identify the possible musculoskeletal causes of compensatory postural arrangements. The functional disability and pain level were assessed before, immediately after and 3 months post-intervention in both groups. Data were analyzed using repeated measures ANOVA. Results: Thirty-five subjects per group participated, with the CPM group averaging 38.6 ± 10.1 years and the CG 40.2 ± 12.1 years. The CPM group experienced significantly greater pain relief both immediately post-intervention and at the 3-month follow-up (*p* < 0.001). While the disability perception decreased in both groups, the CPM group showed superior improvement at the 3-month follow-up (*p* < 0.001). Conclusions: The CPM represents promise for enhancing motor control and quality of life, suggesting potential benefits for other musculoskeletal issues. Future research should explore its broader applications and underlying physiological mechanisms.

## 1. Introduction

Low back pain (LBP) has been the main contributor to the overall burden of musculoskeletal conditions for at least the last three decades [1]. It accounts for 7.7% of the global Years Lived with Disability and is the leading cause of disability in most countries [1,2]. In 2020, it was estimated that 619 million people (95% uncertainty interval 554 to 694) reported having LBP globally [2]. The majority of its social and economic costs are attributable to people with a prolonged disability—suffering from chronic and/or recurring LBP [3]. The prevalence of LBP and its associated healthcare utilization exert a significant social and economic impact that is expected to increase in the coming decades [2,4,5,6]. In particular, the burden on healthcare systems in the five years following the initial diagnosis of individuals with chronic low back pain has recently been estimated in terms of outpatient services, physical therapy, epidural steroid injections and pharmacological treatments (including opioid medications), with costs reaching up to USD 50,501, as reported in a U.S. study [7]. In our opinion, the total disability burden and disease-related costs suggest the need for interventions to promote, maintain or restore the physical and physiological well-being of individuals with LBP.

LBP presents a diagnostic challenge due to the myriad potential causes. Specific aetiologies, which account for less than 15% of all back pain cases, involve identifiable pathophysiological mechanisms [3,8,9]. Most patients, constituting the remaining cases, are often labelled as having ‘non-specific LBP’ (NSLBP) [10].

Several intervention strategies are available to manage LBP by reducing the pain intensity and functional limitations. These include pharmacological treatments, rehabilitation incorporating psychological interventions, education programs and physical exercises [11,12,13]. Among these, physical exercises, particularly targeting flexibility, mobility and core stabilization, play a key role in enhancing muscle function and body posture [13]. Such exercises promote muscle balance and stability, which are essential for supporting the lumbar spine and minimizing strain during movement. Strengthening the core is especially important for improving spinal support and reducing stress on the lower back, while flexibility exercises targeting the posterior chain help to alleviate the muscle tightness and stiffness often associated with NSLBP [13]. Adopting non-invasive tools [14] may be useful for periodic monitoring and providing insights into postural alignment to verify the effect of the intervention. Typically, these types of interventions, although performed in a personalized manner, are not based on a preliminary testing phase aimed at identifying the musculoskeletal causes underlying NSLBP [13]. Additionally, different rehabilitative techniques based on postural re-education programs have been used to manage LBP [15,16,17]. All these techniques have proven beneficial for the study groups in comparison to the control groups regarding the overall effect. However, they show contrasting results in terms of the pain reduction, disability alleviation and enhancement of quality of life.

The Canali Postural Method (CPM) is a kinesiological theoretical and practical postural reprogramming method that aims to understand anatomy-related information (based solely on the muscle component) on the possible causes and origins of compensatory postural arrangements and to intervene on a subject to prevent, reduce or remove these arrangements [18]. The CPM defines a working system with new concepts for the three-dimensional understanding of the causes of possible compensatory postural arrangements and intervenes with a personalized approach through individualized gymnastic exercises avoiding a direct intervention on the inflamed or painful body posture [19,20]. However, its specific effects on managing NSLBP are currently underexplored, highlighting the need for further investigation in this context. Moreover, to the best of the authors’ knowledge, no research has specifically investigated the individualization of exercises through a personalized approach aimed at identifying the potential muscular causes of LBP. This study addresses this gap by integrating the personalized evaluation outlined in the CPM and comparing its effects to generic physical exercises on functional disability and pain levels in individuals with NSLBP. We hypothesized that the CPM, by targeting potential muscular imbalances and compensatory postural arrangements, would lead to greater improvements in pain reduction and functional outcomes compared to generic physical exercises.

The primary aim of this study was to assess the effects of a CPM-based intervention versus generic physical exercises on functional disability and pain levels in individuals with NSLBP. The secondary aim was to explore whether a personalized approach targeting muscular imbalances could provide additional benefits in pain management and functional recovery compared to non-specific exercises.

## 2. Materials and Methods

### 2.1. Study Design and Participants

A quasi-experimental study was designed and conducted between January and December 2022. Participants were allocated using a digital randomizer (Microsoft Excel; Microsoft 2007, Redmond, WA, USA), with an allocation ratio of 1:1 and random block sizes of 2, to either the Canali Postural Method (CPM) or a generic exercise program for the control group (CG), to ensure balance between the groups. The type of randomization was simple random. Randomization was performed immediately upon each participant’s recruitment, ensuring that operators had no foreknowledge of group assignment, which minimized selection bias.

Participants aged ≥18 years were eligible for the study if they had low back pain (LBP) assessed by doctors from different sectors of the public or private service. Exclusion criteria were specific causes of LBP (disc herniation, lumbar stenosis, spinal deformity, fracture and spondylolisthesis), systemic disease (cancer and rheumatologic issues), self-declared psychiatric or mental deficits that prevented the understanding of the instructions given for the execution of the exercises to be performed and having received physiotherapeutic interventions or undergone surgical operations within 6 months prior to baseline assessment. Moreover, subjects were excluded if their pain level impaired their ability to perform gymnastic exercises. The persistence of pain was not considered as a criterion for exclusion.

The trial was conducted in compliance with the principles of the Helsinki Declaration and obtained approval from the Ethical Committee of “Sapienza” University of Rome (deliberation n. 119/2021). All participants provided written informed consent.

### 2.2. Procedure

The trial was conducted at a single fitness center in Naples, Italy, selected for convenience due to its suitable facilities and experience in recruiting participants with non-specific LBP (NSLBP). Staff from the center recruited subjects with NSLBP through social media advertising.

After being verbally informed about the study, participants were randomized either into the CPM intervention or the CG in a single-blind manner, meaning they were unaware of their group allocation.

### 2.3. Interventions

Both the CPM and CG interventions comprised eight one-hour sessions, individually administered with an operator on a one-to-one basis. The frequency was twice weekly for four weeks. Each care provider was exclusively assigned to one trial group to prevent cross-contamination of interventions. An operator with a degree in motor science and 6 years of experience in the CPM operated in the intervention group. An expert in motor science, with 12 years of experience, conducted the exercise program in the CG. Their adherence to the protocol was ensured informally through their expertise and commitment to the study’s objectives. No additional formal monitoring or reinforcement measures were implemented during the trial. It was suggested to each subject in both groups to repeat the exercises proposed in the last session at home for 15 min daily up to the end of the follow-up. This could be a remarkable point for comparison with other studies, as home exercise programs are not regularly prescribed in supervised exercise trials for LBP [21]. However, compliance with the home protocol was not formally measured (there is no consistent method to measure participants’ adherence to home exercise programs in supervised exercise trials for LBP) [21], and it was monitored only through verbal reports from the operators.

For each of the two groups, the exercises were explained during the first session, and participants were trained on how to perform them correctly. The execution of the exercises was adapted to the specific conditions of the subjects. In cases where participants experienced pain, the execution methods were adjusted to be pain-free or at least avoid aggravating pain. Standardization was ensured by adhering strictly to the principles and protocols of each method, which include tailored exercises based on participants’ baseline evaluations and physical capacities.

### 2.4. CPM Intervention

The translation of the CPM [18] into a more formal structure to promote its standardized adoption is reported in the Supplementary Materials.

In alignment with the concepts and features of the CPM working system, a set of exercises, including those listed in the postural evaluation phase paragraph, were proposed to the subjects in the CPM group. The CPM, in the initial phase, aims at realigning postural asymmetries and involves the active or passive application of these kinesiological exercises in isometric mode. Attention was focused on stretching the resistant muscles that could prevent the activation of the CPM technical barycentres and selecting the proper angles of application of the exercises to prevent the dominant muscles from intervening (for example, the inhibition of abdominal muscles when performing the exercise described in Supplementary Materials Table S4—2A—due to hamstring resistance or quadriceps dominance). All the proposed exercises took into account the involvement of the movements of the CPM starter joints in terms of the load induced on the muscles involved (in terms of activation, resistance or dominance). The selection of the proposed exercises was personalized, taking into consideration specific postural asymmetries along with other parameters, such as pain intensity, athletic age of the subject, their ability to activate the CPM technical barycentres and the flexibility and range of motion (ROM) of the muscle chains (refer to the paragraph on the postural evaluation phase).

For the CPM group, the first session also served as a test phase to identify the causes and origins of compensatory postural arrangements and the appropriate exercises. During this initial session, exercises were performed in an alternating sequence of strengthening and stretching exercises. The sequence was repeated in sets. In subsequent sessions, the strengthening exercises were progressively increased in duration, tailored to the participants’ abilities, which resulted in a reduction in the number of sets.

### 2.5. Control Group

In the absence of a definitive gold standard intervention for exercises for LBP treatment [13], the CG underwent a program of general exercises, administered individually, aimed at lengthening and strengthening the posterior and anterior chains as well as the adductors and at strengthening the core to provide relief from NSLBP.

The program consisted of bridging exercises to mobilize the pelvis and spine while strengthening the gluteus and hamstring muscles. Additionally, hamstring stretches were incorporated to mobilize the spine and lengthen the muscles of the posterior chain. Supine cycling was employed to strengthen the abdominal muscles and coordinate the anterior and posterior lumbar muscles. Lower abdominal crunch exercises in the supine position aimed to strengthen the abdominal muscles while maintaining a flat back on the mat. Prone plank exercises were utilized for abdominal muscle strengthening, and seated inner thigh stretch exercises were performed to lengthen the adductor muscles of the hip. Lastly, kneeling exercises were implemented to lengthen the quadriceps.

In the control group, the session was divided into an initial phase of stretching exercises (10 min), a central phase of strengthening exercises (40 min) and a final phase of stretching exercises (10 min). The exercise sequence was repeated in sets. In the CG, the approach to strengthening exercises in subsequent sessions mirrored that of the CPM group.

### 2.6. Measurements

The primary outcome measured the perceived level of disability due to LBP using the Roland and Morris Disability Questionnaire (RMDQ), a self-administered evaluation scale. The RMDQ is validated in Italy [22] and consists of 24 items, with higher scores on the 24-point scale indicating greater levels of disability [23]. The RMDQ has been shown to yield reliable measurements, which are valid for inferring the level of disability, and to be sensitive to change over time for groups of patients with LBP [24].

Secondary outcome measures included the assessment of lumbar physical discomfort using a 100 mm Visual Analogue Scale (VAS), where scores ranged from 0 (no pain) to 100 (the worst possible pain). The VAS has demonstrated reliability and satisfaction in pain measurement [25].

Outcome assessments were conducted at baseline, post-intervention and at a follow-up of 3 months.

### 2.7. Blinding

Blinding was not feasible in this study due to the nature of the interventions. Each operator was an expert in their respective method and had a vested interest in applying the interventions to the best of their ability. As a result, the operators were not blinded to the group allocation of participants.

To limit potential bias, several steps were implemented. First, all outcome assessments were self-reported by the participants, reducing the possibility of operator influence on the reported outcomes. Additionally, the statistical analyses were conducted independently by one of the authors, who was not involved in the delivery of the interventions. This helped ensure objectivity and further reduced the potential for bias in the analysis.

### 2.8. Sample Size

The sample size calculation was based on a priori calculation with Gpower (v 3.1) [26]. To find a significant effect size of 0.25 with a power of 0.80, and an α of 0.05, 44 participants were needed to complete the study [27]. 

### 2.9. Statistical Analysis

Normality has been tested by evaluating Skewness and Kurtosis for the key variables of the study. All the variables showed values between −2 and +2, confirming a normal-like data distribution [28]. Baseline descriptive statistics were reported for both groups, including personal and outcome measures. Continuous data were expressed as means and standard deviations (SDs), while categorical data were presented as absolute numbers and percentages (%). Levene’s test was used to assess the homogeneity of variances. Baseline characteristics of the CPM group and CG were compared using Student’s *t*-test for continuous variables and the chi-squared test for categorical variables. Mauchly’s test was conducted to assess the assumption of sphericity, and repeated-measures ANOVA was performed to compare mean scores of each outcome variable across different assessment time points and between groups. Post hoc multiple comparisons were adjusted using Tukey’s correction. All analyses were performed using R software (version 3.6.3; R Core Team, 2020), with significance set at *p* < 0.05.

## 3. Results

### 3.1. Recruitment

In total, 70 participants successfully entered the study. Of these, 35 subjects were randomly assigned to the Canali Postural Method (CPM) program, while the remaining 35 were assigned to the control group (CG) program. No drop out was experienced.

### 3.2. General Characteristics and Homogeneity of Participants

Participants’ characteristics were similar across groups at the baseline (see Table 1).

### 3.3. Effects of the Intervention

Mauchly’s test of sphericity was not significant (W = 0.942, *p* = 0.133). However, since Levene’s test for homogeneity of variances showed a significant result at one of the three time points for the Roland and Morris Disability Questionnaire (RMDQ) post-intervention (*p* < 0.05), a robust ANOVA model was adopted.

**Figure 1:**
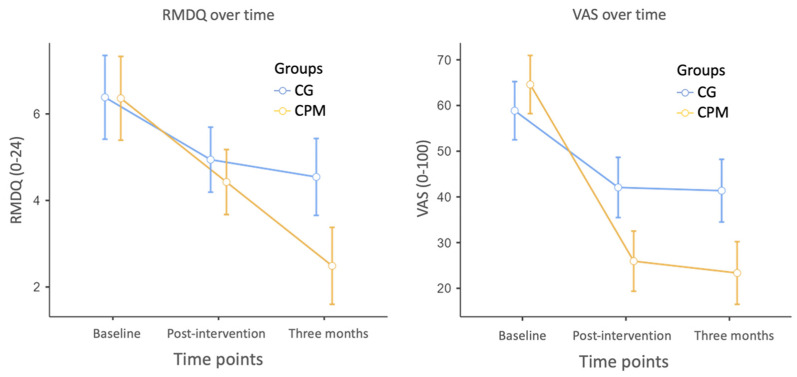
Graphical representation of ANOVA analysis. Legend: RMDQ = Roland and Morris Disability Questionnaire and VAS = Visual Analogue Scale. Error bars represent confidence intervals.

The perceived level of disability due to LBP using the RMDQ and the level of pain using the Visual Analogue Scale (VAS) in the three time points are reported in Table 2 and Figure 1.

The results of the ANOVA for the RMDQ indicated a significant GROUP x TIME interaction effect (F(2, 136) = 3.32, *p* = 0.039, η^2^p = 0.047), suggesting that the changes over time differed between groups. Additionally, the within-subject effects revealed a significant main effect of TIME (F(2, 136) = 24.38, *p* < 0.001, η^2^p = 0.264), indicating a change in RMDQ scores over time. For the between-subject effects, the main effect of GROUP was significant (F(1, 68) = 4.73, *p* = 0.033, η^2^p = 0.065), indicating a difference between the two groups across time points. Post hoc analyses are presented in Table 3. Pairwise comparisons using Tukey’s correction revealed several significant differences. Within the CPM group, scores significantly decreased between baseline and post-intervention (t = 3.30, *p* = 0.019), baseline and three months (t = 6.08, *p* < 0.001) and between post-intervention and three months (t = 3.77, *p* = 0.005). Between the CPM group and CG, a significant difference was found at three months (t = 3.3, *p* = 0.021).

With respect to the VAS, the ANOVA revealed a significant GROUP × TIME interaction effect (F(2, 136) = 9.11, *p* < 0.001, η^2^ = 0.118), as well as significant main effects for both GROUP (F(1, 68) = 9.75, *p* = 0.003, η^2^ = 0.125) and TIME (F(2136) = 57.02, *p* < 0.001, η^2^ = 0.456). Given the significant interaction effect, pairwise comparisons using Tukey’s correction were performed. The post hoc analysis (Table 4) indicated that within the CPM group, scores significantly decreased between baseline and post-intervention (t = 9.70, *p* < 0.001) and between baseline and three months (t = 8.97, *p* < 0.001). The CG also showed a significant reduction between baseline and post-intervention (t = 4.22, *p* = 0.001) and between baseline and three months (t = 3.80, *p* = 0.004), although the effect was smaller than in the CPM group.

Between the CPM and CG, a significant difference was found both at post intervention (t = 3.45, *p* = 0.012) and at three months (t = 3.7, *p* = 0.006).

## 4. Discussion

### 4.1. Main Findings

This study demonstrated that the Canali Postural Method (CPM), a kinesiological approach to posture reprogramming, yielded superior mid-term efficacy in reducing low back pain (LBP) intensity and functional disability levels compared to generic strengthening, stretching and core-based exercises selected for pain relief.

While both groups experienced pain alleviation, the Canali Postural Method (CPM) group exhibited a more significant reduction both post-intervention and at the 3-month follow-up. Specifically, the CPM group showed a Visual Analogue Scale (VAS) reduction from 64.6 to 23.4 mm, an absolute change of 41.2 mm, which exceeds the established Minimal Clinically Important Difference threshold of 15 mm for NSLBP [29]. This confirms that the observed pain reduction is not only statistically significant but also clinically meaningful. Additionally, the functional disability decreased in both groups post-intervention, but notably with better results at the 3-month follow-up in the CPM group.

Moreover, both groups showed high compliance, with a 100% attendance rate at scheduled appointments. Although the adherence to the home protocol was not formally measured (there is no consistent method to measure participants’ adherence to home exercise programs in supervised exercise trials for LBP) [21], it was monitored through verbal reports from the operators. Both groups achieved satisfactory results in the assessment conducted after the conclusion of the training program. The ability to exercise without pain and the rapid functional improvement likely acted as motivational factors contributing to compliance.

### 4.2. Comparison with Previous Research

Our findings align with those of a recent study in which subjects with chronic LBP underwent an 8-week intervention with either progressive postural control or core stability exercises [30]. Similarly to our results, both groups experienced significant reductions in pain intensity and functional disability, though a slight return to baseline conditions was observed at the 6-month follow-up. However, our follow-up period was shorter, making a direct comparison challenging.

Additionally, our results align, particularly regarding pain levels, with those reported in a systematic review involving subjects with NSLBP undergoing hip strengthening exercises [31]. While a greater effect of the exercise program on functional disability was reported, the study populations had more severe starting conditions and participated in a greater number of exercise sessions compared to our study.

Regarding specific interventions such as Pilates, yoga and others, a recent review concluded that no single method can currently be considered superior to another [32]. Our findings align with those of similar studies cited in a systematic review [33]. However, it is important to note that in these studies, the personalization of exercises is either not mentioned [34,35,36,37] or only broadly stated with phrases such as “physiotherapists personalized the exercises based on a preliminary assessment” [38,39,40]. These descriptions lack detailed evaluative processes, making them difficult to compare methodologically with the structured approach outlined in our Supplementary Materials for the CPM.

### 4.3. Study Limitations

This study has several limitations. The recruitment via social media may have introduced a selection bias, as participants with an interest in fitness or social media engagement might not fully represent the general population with NSLBP. While the sample size was adequately powered for the medium effect size, it remained relatively small and was composed exclusively of individuals with NSLBP, limiting the generalizability. Psychiatric and cognitive impairments were assessed based on participants’ understanding of exercise instructions, relying on self-reports and observational judgment. The CPM program included a postural evaluation phase, leading to differences in personalization between groups, which may have contributed to the superior outcomes or may have led to greater perceived relevance, motivation and engagement among participants in the CPM group. Future studies should standardize the degree of personalization across intervention arms or include a control group with personalized exercises to better isolate the specific effects of the CPM intervention. Although supervised sessions were provided, the home exercise adherence was not objectively monitored, potentially affecting the outcomes. Operators’ verbal reports offered some indication of compliance, but the lack of a standardized tracking method remains a limitation, which is compounded by the absence of consistent strategies for monitoring the home exercise adherence in supervised LBP trials. Future studies should integrate objective tracking tools, such as wearable monitors, exercise logs or digital applications, to improve the adherence assessment. The 4-week intervention, though practical, was shorter than in many similar studies, and the 3-month follow-up, while sufficient for short-term effects, may not fully capture the long-term impact. A longer follow-up would introduce potential confounding factors (e.g., trauma recurrences, lifestyle changes and additional treatments) and increase dropout risk, potentially affecting data reliability. Future research should consider larger sample sizes to support extended follow-ups and better assess intervention persistence. The blinding of operators was unfeasible due to the nature of the interventions, posing a potential source of bias despite mitigation efforts, such as self-reported outcomes and independent statistical analysis. As experts in their respective interventions, operators may have unconsciously influenced participants through subtle behaviors or attitudes.

Lastly, this study relied exclusively on self-reported questionnaires, which, while validated, lack objective assessment. Future research should incorporate instrumental measures, such as tensiomyography, algometry and electromyography, to provide more objective evaluations of intervention effects.

### 4.4. Potential Explanations for Findings

The CPM emerges as a novel perspective within the field of kinesiology, aiming to integrate and adapt kinesiological methodologies for posture assessment and posture reprogramming. Initially conceived as a professional practice, the current work marks its transition into a formalized methodological framework that needs an extensive discussion within the scientific community. As part of this transition, a pilot efficacy study has been conducted to provide insights into the practical application of the method and bolster the formalization process. Moving forward, collaborative efforts and critical evaluation will be essential in further refining and advancing the CPM within the field of kinesiology. It is worth noting that, as reported in the Supplementary Materials, the inter-rater reliability and internal consistency were previously assessed for the analysis of reference points—the only operator-dependent element of the assessment phase—yielding excellent results.

The observed efficacy of the CPM may be linked to its methodological approach, which prioritizes neuromuscular adaptation and postural control rather than simply strengthening or stretching the muscles involved. By fostering the development of new motor patterns and a more harmonized movement experience, the CPM may enhance motor control and reduce musculoskeletal stress, contributing to pain relief and functional improvement. During dynamic movements, particularly in sport-specific transitions between a 90-degree position and full extension, abdominal muscles may be insufficient—especially in the eccentric phase—to counteract the peripheral resistance due to intrinsic weakness or the dominance of peripheral muscles. This imbalance can lead to compensatory postural adjustments, increasing the risk of musculoskeletal disorders like low back pain. The CPM, which differs from standard core strengthening, addresses these imbalances by targeting opposing chains—particularly of the second and third degree (see Supplementary Materials)—and integrating isometric exercises at critical angles, improving the motor control in movements requiring these specific positions.

Beyond its clinical implications, the CPM may also have broader societal relevance. Given the increasing burden of LBP, which is projected to rise by 36.4% globally by 2050 [2], effective non-pharmacological approaches could help reduce healthcare costs, the loss of productivity and the reliance on surgical procedures or opioid prescriptions, which carry significant risks of addiction and overdose [41,42]. Furthermore, from a psychological and socio-humanistic perspective, posture is deeply linked to self-perception and social interactions. As highlighted by Topino et al. [43], posture influences assertiveness, self-efficacy, self-esteem and overall well-being. In this context, the CPM can play a crucial role in shaping a constructive postural attitude.

### 4.5. Future Directions

Overall, the CPM shows promise in improving posture among subjects with NSLBP, potentially fostering new movement and postural patterns that reduce musculoskeletal strain and enhance quality of life. However, further evaluation is required to strengthen the evidence supporting its efficacy. In particular, additional research is needed to refine its neuromuscular foundations and optimize individualization. Investigating neuromuscular adaptations through surface electromyography and motion analysis could help elucidate its underlying mechanisms. Moreover, randomized controlled trials with larger and more diverse samples are needed to establish stronger evidence of effectiveness. Long-term follow-up studies should be designed to assess the persistence of the CPM’s benefits and explore strategies to enhance adherence. Expanding the CPM’s application to other musculoskeletal conditions and integrating technological tools for personalized implementation also represent promising avenues for investigation.

## 5. Conclusions

The formal structuring of the CPM represents an interesting advancement for the method itself, as it fosters exchanges and discussions within the scientific community. Standardizing its application ensures consistency in training and implementation across practitioners, facilitating rigorous research and evaluation, leading to a deeper understanding of its mechanisms and efficacy.

By integrating postural assessment and neuromuscular adaptations, enhancing proprioceptive awareness, correcting muscle imbalances, strengthening technical barycentres and improving motor coordination, the CPM offers a promising approach to bolstering motor control and, consequently, improving quality of life. We believe that the CPM approach has the potential to significantly contribute to the ongoing debate on the reading and reprogramming of human postures within the kinesiological domain and, potentially, in strict collaboration with trained healthcare personnel, in the context of exercise therapy.

Several directions for future research can be pursued: exploring the CPM’s potential for addressing musculoskeletal issues beyond those leading to NSLBP; conducting mechanistic studies to understand the underlying physiological mechanisms comprehensively; and developing supporting tools for the smoother application of the method.

## Figures and Tables

**Table 1:** Baseline characteristics of subjects.

Characteristics	CPM (n = 35)	CG (n = 35)	Statistics	*p*-Value
Age (yr), mean ± SD	38.6 ± 10.1	40.2 ± 12.1	0.60 *	0.55
BMI, mean ± SD	22.6 ± 2.9	23.1 ± 3.1	0.69 *	0.483
Female, n (%)	19 (54.2%)	18 (51.4%)	0.12 #	0.902
RMDQ (0–24), mean ± SD	6.4 ± 2.8	6.4 ± 3.0	0.03 *	0.973
VAS (0–100), mean ± SD	64.6 ± 18.0	58.9 ± 19.8	−1.27 *	0.209

BMI: Body Mass Index; CPM: Canali Postural Method Group; CG: control group; RMDQ: Roland and Morris Disability Questionnaire; SD: standard deviation; and VAS: Visual Analogue Scale. * *t*-test; # chi-square.

**Table 2:** Comparisons between experimental and control groups on RMDQ and VAS in different time points.

**Variables**	**TIME**	**CPM** **M ± SD**	**CG** **M ± SD**	t *	**SE**	**95% CI**	**d**
RMDQ (0–24)	Baseline Post-intervention 3 months	6.36 ± 2.76 4.43 ± 1.69 2.49 ± 2.34	6.38 ± 2.98 4.94 ± 2.66 4.54 ± 2.90	0.03 0.97 3.26 *	0.69 0.53 0.63	−1.35, 1.39 −0.55, 1.58 0.80, 3.31	0.00820 0.23208 0.77948
VAS (0–100)	Baseline Post-intervention 3 months	64.59 ± 17.95 25.94 ± 18.92 23.35 ± 18.25	58.87 ± 19.77 42.07 ± 20.13 41.36 ± 22.24	−1.27 3.45 * 3.70 *	4.51 4.67 4.86	−14.77, 3.28 6.81, 25.45 8.31, 27.71	−0.30341 0.82571 0.88538

NOTES: RMDQ = Roland and Morris Disability Questionnaire; VAS = Visual Analogue Scale; CPM = Canali Postural Method Group; CG = control group; M = mean; SD = standard deviation; t = t-value from *t*-test with Tukey’s correction; SE = Standard Error; CI = confidence intervals; d = Cohen’s d; and * *p* < 0.05.

**Table 3:** Post hoc multiple comparisons for RMDQ.

Time	Group	Time	Group	Mean Difference	SE	t	p_tukey_
T_0_	CG	T_0_	CPM	0.0235	0.686	0.0343	1.000
		T_1_	CG	1.4418	0.587	2.4555	0.152
		T_2_	CG	1.8411	0.637	2.8897	0.056
	CPM	T_1_	CPM	1.9354	0.587	3.2961	0.019
		T_2_	CPM	3.8728	0.637	6.0788	<0.001
T_1_	CG	T_1_	CPM	0.5171	0.533	0.9709	0.926
		T_2_	CG	0.3993	0.514	0.7761	0.971
	CPM	T_2_	CPM	1.9374	0.514	3.7658	0.005
T_2_	CG	T_2_	CPM	2.0553	0.630	3.2608	0.021

RMDQ = Roland and Morris Disability Questionnaire; T_0_ = baseline; T_1_ = post-intervention; T_2_ = follow-up, 3 months post intervention; CG = control group; CPM = Canali Postural Method group; mean difference = difference in means between the compared groups/time points; SE = Standard Error; t = t-value from *t*-test; and p_tukey_ = adjusted *p*-value of *t*-test from Tukey’s post hoc test.

**Table 4:** Post hoc multiple comparisons for VAS.

Time	Group	Time	Group	Mean Difference	SE	t	p_tukey_
T_0_	CG	T_0_	CPM	−5.729	4.51	−1.269	0.800
		T_1_	CG	16.800	3.99	4.216	0.001
		T_2_	CG	17.502	4.60	3.805	0.004
	CPM	T_1_	CPM	38.657	3.99	9.701	<0.001
		T_2_	CPM	41.242	4.60	8.965	<0.001
T_1_	CG	T_1_	CPM	16.129	4.67	3.454	0.012
		T_2_	CG	0.702	4.50	0.156	1.000
	CPM	T_2_	CPM	2.585	4.50	0.574	0.992
T_2_	CG	T_2_	CPM	18.011	4.86	3.704	0.006

VAS = Visual Analogue Scale; T_0_ = baseline; T_1_ = post-intervention; T_2_ = follow-up, 3 months post-intervention; CG = control group; CPM = Canali Postural Method group; mean difference = difference in means between compared groups/time points; SE = Standard Error; t = t-value from *t*-test; and p_tukey_ = adjusted *p*-value of *t*-test from Tukey’s post hoc test.

## Data Availability

The data presented in this study are available on request from the corresponding author due to privacy reasons.

## Supplementary Materials

The following supporting information can be downloaded at https://www.mdpi.com/article/10.3390/healthcare13080869/s1. The Canali Postural Method [18,44,45,46,47,48,49,50,51,52,53,54,55,56,57,58,59,60,61,62,63,64,65,66,67,68,69].

## Abbreviations

The following abbreviations are used in this manuscript:

BMI	Body Mass Index
CG	Control group
CPM	Canali Postural Method
LBP	Low back pain
NSLBP	Non-Specific low back pain
RMDQ	Roland and Morris Disability Questionnaire
SD	Standard deviation
SE	Standard Error
VAS	Visual Analogue Scale
