# Supplementary material for: A Quasi-Experimental Controlled Study to Evaluate the Effects of a Kinesiologic Approach—The Canali Postural Method—To Posture Reprogramming for Non-Specific Low Back Pain
Source: Healthcare (Basel). 2025 Apr 10;13(8):869. doi: 10.3390/healthcare13080869 (PMC12026616; doi:10.3390/healthcare13080869)
Supplement: Supplementary file 1 [file healthcare-13-00869-s001.zip › healthcare-3481819-supplementary.pdf]

SUPPLEMENTARY MATERIAL

CONTENT

THE CANALI POSTURAL METHOD 2

1. Background 2
2. The Basic Idea 3

The CPM working system 3

The CPM methodology – postural assessment phase 12

The CPM methodology – postural reprogramming phase 25

3. References 27

THE CANALI POSTURAL METHOD

1. Background

Body posture refers to the alignment and connections of various body parts, allowing for a desired stance and anti-gravity function [44–46]. Dynamic control of joint stiffness maintains balance and facilitates voluntary movements [46].

Poor posture can arise from limited range of motion (ROM) in joints, or from weak or tight muscles [47,48]. The persistence of these limitations, in fact, can induce compensatory postural arrangements to reduce the demand on the more distressed body segments [49]. These arrangements, in the long term, could lead to more stable posture faults (e.g. lumbar hyperlordosis, thoracic hyperkyphosis, protracted shoulders, protruded head) [50–52] or pain [53], and, in case of reiterated critical movements, even injuries [54]. Posture is not merely a prerequisite for movement but also significantly impacts overall health and well-being. In fact, it can adversely affect the cardiovascular and autonomic nervous systems, leading to decreased stroke volume, increased heart rate, and relative activation of sympathetic tone [55]. Moreover, bad posture can also result in chronic primary headaches, reduced respiratory function [56,57], impaired blood circulation [58], and a range of other issues, including digestive problems [59]. Additionally, it may have detrimental psychosocial effects, influencing mood, confidence, self-esteem, and resilience to stress [60].

Several factors can contribute to a limited ROM in joints, or to weak or tight muscles, including a sedentary lifestyle, poor ergonomic setup, lack of awareness, ageing, psychological factors such as stress and anxiety, as well as injury, trauma, or medical conditions [61,62]. Nevertheless, individuals can improve their posture through deliberate practice and corrective measures. Through specific exercises and ergonomic adjustments, they can gradually improve their postural habits and alignment [63,64]. Within clinically controlled and stabilized health conditions, Kinesiology, the science of human movement, focuses on preventing injuries and functional recovery post-rehabilitation [65]. Kinesiology works through exercise programmes to promote the strength, endurance and flexibility of the muscles responsible for supporting good posture. Kinesiology combines a range of knowledge

sources, incorporating insights from participation in physical activities, along with professional practices dedicated to physical activity [66].

A kinesiological approach to posture reprogramming is the Canali Postural Method (CPM) [18]. It originated as a professional practice based on the direct observation of thousands of subjects over 35 years of experience and discussions with kinesiologists and is now evolving into a more structured methodology [18].

In the following sections, the formal structure of CPM is described for its standardized adoption.

2. The Basic Idea

The Canali Postural Method (CPM) is a theoretical and practical postural reprogramming method that aims to understand anatomy-related information (based solely on the muscle component) on the possible causes and origins of compensatory postural arrangements, and to intervene on a subject to prevent, reduce or remove these arrangements. The CPM intervenes with a personalized approach through individualized gymnastic exercises avoiding direct intervention on inflamed or painful regions [18]. It assumes a prerequisite: the synergistic relationships among muscles, due to their role in enabling and regulating joint mobility and stabilizing body position, should be assessed and enhanced through static exercises based on the demands of dynamics, as also observed by Oba and colleagues [67]. Once the static postural prerequisite has been guaranteed, the specific needs in terms of joint angles necessary in target motor gestures, from everyday life to elite sport, can be considered to complete customized programmes that specifically expand the set of static exercises and introduce other dynamic ones (this part is not covered within the present work).

Since there are numerous possibilities to create static postural prerequisites, the CPM defines a working system with new concepts for the three-dimensional understanding of the causes of possible compensatory postural arrangements.

The CPM working system

Concepts

The working system proposed in the CPM considers the following concepts.

1) STARTER JOINTS – the coxofemoral (hip), tibiotarsal (ankle) and gleno-humeral (shoulder) joints are defined as ***starter joints*** since their movements in the sagittal plane (flexion and extension), unlike all the other joints (defined as *non-starter joints*), activate other synergistic muscles with respect to agonist and antagonist muscle groups.
2) OPPOSED CHAINS – Within the Steindler concept of a kinetic chain as 'a combination of several successively arranged joints constituting a complex motor unit' [68], CPM refers to an *acting chain* as the part that initiates a specific movement, thus causing flexion within this chain. In contrast, within the same movement, CPM defines the ***opposed chain*** as the complementary part that is stimulated to extend and resists extension, facilitating motion control. Three chains are defined:
    a. ***1st degree opposed chain*** – This is the relationships between the action (contraction or elongation) of a muscle near a non-starter joint and the action near a starter joint (***opposed head*** acting as the opposed chain). For example, when a non-starter joint flexes and the muscle region closer to this joint is required to elongate (e.g. quadriceps in knee flexion), the opposed head must also elongate to prevent impact on other muscle groups involved in stabilizing the starter joint and/or to avoid compensations. If compensations occur at the starter joint despite an opposed head capable of elongating, the cause should be investigated in the 2nd or in the 3rd degree opposed chains (as explained further).
    b. ***2nd degree opposed chain*** – The attention is on a kinetic chain that crosses a starter joint when the subject has hip extension (>0°). For example, i) the same movement as in the previous point with an unwanted implication of the hip, or a kicking leg when considering the ii) charging phase – knee flexion with hip extension – and iii) the releasing phase – up to the knee extension with the hip to be kept extended as long as possible before flexing. The 2nd degree analysis investigates whether all the elements of the part of the kinetic chain stimulated to extend and resist extension are functioning

correctly. Three exemplificative situations related to the above reported examples are: i) in a knee flexion performed while resisting an external force, the eccentric contraction of quadriceps could cause an early hip anteversion if the abdominal muscles are weak; ii) in the charging phase of the kicking leg, knee flexion should be performed to avoid pelvis anteversion. This could be caused either by rigid antagonist muscles of hip extension or by weak abdominal muscles unable to contract sufficiently eccentrically; iii) during the release phase, the anteversion of the hip induced by the contraction of the quadriceps should be avoided to protect the spine. This is achieved through eccentric contraction of the hip extensors and abdominal muscles, acting as stabilizers of the pelvis.

c. ***3rd degree opposed chain*** – Similarly to the 2nd degree opposed chain, this focuses on a kinetic chain that involves a starter joint, but in this case, with the subject having the hip flexed (90°). In this body position, the analysis investigates the contraction capacity of the anterior chain (acting chain) in comparison to the capacity and mode of extension of the posterior chain (opposed chain) in resisting this contraction.

3) FLEXION–EXTENSION MUSCLE RELATIONSHIP – CPM defines the ***flexion–extension relationship*** as the capacity of contraction of the acting chain in comparison to the capacity and mode of extension of the opposed chain in resisting this contraction. Insufficient flexibility in one muscle group of the opposed chain can result in inappropriate activation of the acting chain (e.g. in a supine decubitus position with the lower limbs flexed at an angle of 90 degrees, hamstring rigidity can inhibit abdominals and lead to inappropriate quadriceps activation) leading to compensations (e.g. lumbar hyperlordosis). The analysis of the opposite chains (in the three degrees reported above) helps to identify where and why the flexion–extension relationship may be incorrect. It is essential to verify correct flexion–extension muscle relationships dynamically, considering challenges posed by changes in body positions (from hip extended to hip flexed and vice versa).

Features

The working system proposed in the CPM considers the following six interlinked features.

1) In movements involving the forward translation of the pelvis along the sagittal axis (e.g. in walking and running), it is also important to consider its simultaneous tilt along the transverse axis (i.e. in the sagittal plane). The former induces sagittal solicitations in the spine and a tendency toward lumbar hyperlordosis, necessitating stabilization strategies. A pelvis tilt along the transverse axis brings back the lumbosacral spine. The described simultaneous translation and tilt are referred to as the **bi-axial movement of the pelvis (roto-translation)**. Moreover, moving the lower rib cage backward allows for pushing back the thoracolumbar spine. The lower ribcage retraction can be improved by abdominal muscles. In fact, the rectus abdominis, in addition to caudally depressing the ribcage, decreases its anteroposterior diameter [69]. This effect, achievable due to the positioning of insertions and origins of the rectus, is obtainable only through a backward pull of the ribcage. Although to a lesser extent, this action necessarily pushes back the lower thoracic spine and, consequently, the entire thoracolumbar tract. The simultaneous bi-axial movement of the pelvis and lower ribcage retraction position the subject as shown in Figure S1, creating a force couple that enables the spine to resist dynamic solicitations and avoid hyperlordosis.

Figure S1. Schematic representation of forces acting on the spine in movements that involve forward translation of the pelvis along the sagittal axis

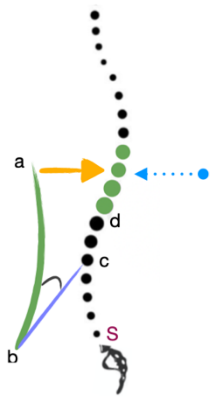

Legend: a) insertion of abdominal muscles (ibcage); b) origin of abdominal muscles (pelvis) and insertion of iliopsoas muscle (lesser trochanter of femur); c) origin of iliopsoas muscle (vertebrae); d) transition between thoracic kyphosis and lumbar lordosis.

2) **Two body positions, hip flexed (90°) and hip extended (>0°),** which in a dynamic context are determined by the above-described bi-axial movement of the pelvis, **should both be reached and maintained as a postural prerequisite for full mobility**. In these positions, it is important not to overload potentially compensatory areas and to maintain the physiological curves of the spine. Three muscle groups play a key role in achieving and maintaining these positions and are subjected to the most stress; for these reasons these muscles are defined by the CPM as ***technical barycentres***:

   a. ***Abdominal muscles*** – In addition to their role in stabilizing the thoracolumbar spine, as described earlier, the abdominal muscles also contribute to the stabilization of hip extension. They counteract the forward translation of the hip, producing an 'elastic effect' that induces an 'auxotonic' contraction in the hip extensor muscles.

   b. ***Hamstring muscles*** – In the forward bi-axial movement of the hip described in the first feature, the hamstrings, along with the gluteus maximus, are the primary hip extensors. If the hamstrings are not activated, a postural arrangement occurs, resulting in pelvic anteversion. In this situation, hip extension is primarily carried out by the gluteus and lumbar muscles, leading to an increased load on the lumbar spine.

   c. ***Scapular stabilizer muscles*** – In shoulder flexion, the gleno-humeral joint requires the stabilization of the scapulae in adduction to work in synergy with the abdominal

muscles. In fact, scapular stabilizer muscles have the same working direction as abdominal muscles in relation to pectoral and lumbar muscles.

3) In the movements that involve **inversions of direction** (for example the step, with the anteroposterior oscillations of the legs), it is necessary to stabilize the intermediate static points at the inversion of the motion through the correct activation of technical barycentres involved in the two phases.
4) The movement of the joints along their ROM, even when these are the fulcrum of a lever where a force is applied, produces **peripheral muscular resistances** that can act against the technical barycentres, also inducing their deactivation.
5) With a more comprehensive approach than focusing solely on specific agonist/antagonist relationships, the movements of the joints are connected in a **circular system**. In this system, each flexion–extension muscle relationship, influenced by the strength and resistance expressed, has an impact on the functioning of the entire kinetic chain. With the circular system approach, in the two key body positions (hip flexed and hip extended) determined by the bi-axial movement of the pelvis, the kinetic chain is challenged by a possible excess of strength (dominance) or resistance of a muscle with respect to the technical barycentres, or by a lack of stabilization due to insufficient activation of the latter. This leads to compensatory postural arrangements in other parts of the chain with a possible onset of pain and trauma in such parts. When the activation of technical barycentres exerts greater force than that opposing the joints, the entire system achieves balance. A significant movement illustrating this is the step, whether in walking or running, involving physiological movements of the trunk in the three planes: advancement in the sagittal plane; rotation in the transverse plane; and displacements in the frontal plane. The support phase is stabilized by the sagittal eccentric action of the abdominal muscles in correspondence with the supporting limb. The alternation of the supporting limb combines part of the anterior chain on one side with part of the posterior chain on the other (anterior-left with posterior-right chains when it is the left limb that supports

the step, and vice versa). In case of dominance, resistance to abdominal muscles, or insufficient activation of this technical barycentre, compensatory postural arrangements of the body occur unknowingly across the three planes, potentially forcing physiological curves of the spine under excessive loads due to misalignment. Furthermore, these compensatory postural arrangements of the body can lead to a reduction in the ROM of starter joints, imposing excessive loads on related muscles or tendons. Consequently, this can result in pain in the compensation zone, additional compensatory arrangements and decreased ROM in other joints involved in the kinetic chain in an iterative manner (see Figure S2a). CPM considers that, in the absence of a direct trauma, these signs are often related to overloads due to inhibition of functionality in other parts of the body.

6) With specific reference to the step, the aforementioned dominance or resistance regarding the abdominal muscles or their insufficient activation can be detailed within side-dependent (in the frontal plane) cycles of postural compensations (see Figure S2b). The relative weakness of abdominal muscles can be more pronounced on one side, defined as the ***weak side***. Muscular resistance can be accentuated on one side (the left or right), termed the ***rigid side***. The presence of a weak and / or rigid side determines a limitation of availability in the intended movement (for example, a limited ROM of the right ankle causes a reduced extension of the left leg). This limitation, in turn, can lead to an imbalance in loading time, with one side working more than 50%, defined as the ***loaded side***, (using the same example, the left limb might anticipate its movement and spend more time than the opposite one during the supporting phase, making the left side the loaded side). This uneven involvement of limbs causes greater stress on one side of the body, termed the ***distressed side***, with the possible onset of tensions, pain and further compensatory arrangements. These, cyclically, may impact dominance and / or resistance.

Figure S2. Compensatory cycle (a) and CPM sides as a conceptual framework on the layers of the compensatory cycle (3D schema)

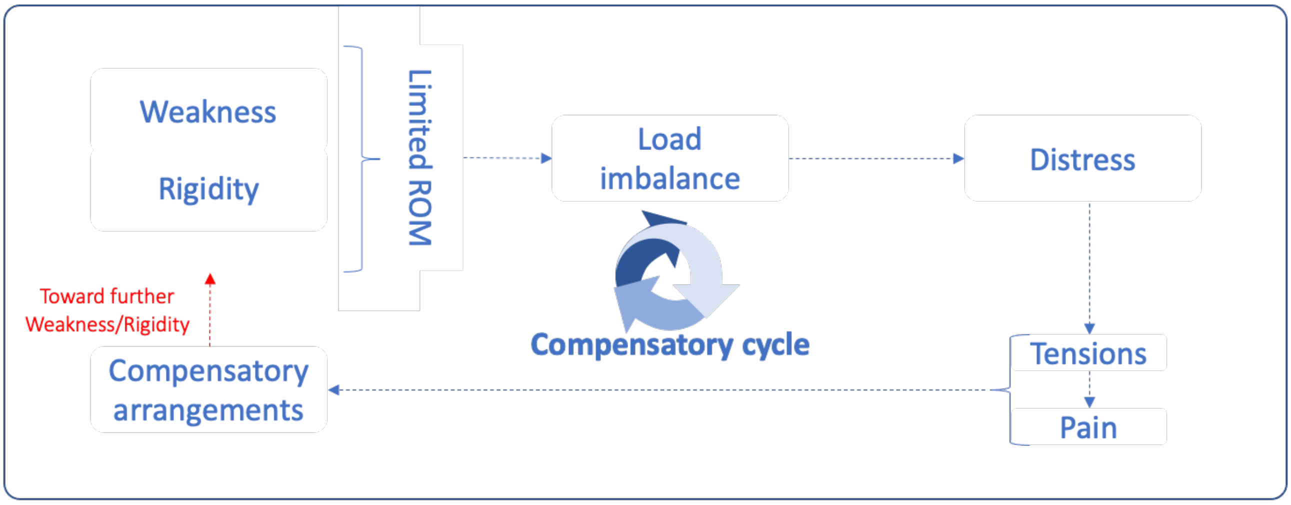
Weakness
Rigidity
Limited ROM
Load imbalance
Distress
Compensatory cycle
Toward further Weakness/Rigidity
Compensatory arrangements
Tensions
Pain

Figure S2A

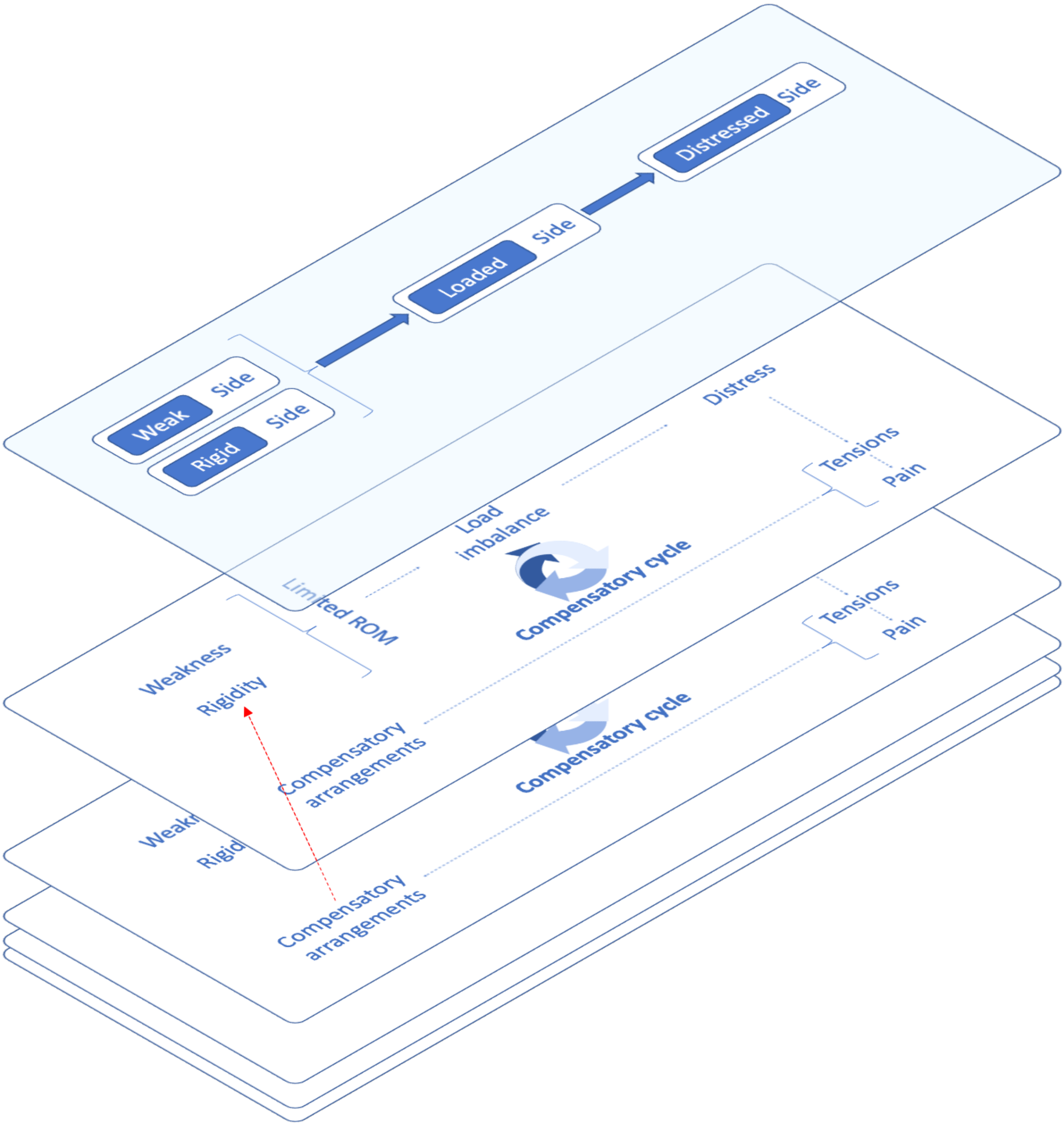

Figure S2B

Legend: Figure S2A schematically illustrates all the steps of the (clockwise) cycle that begins with muscle weakness or rigidity and leads to compensatory adaptations. These adaptations may cause further (red arrow) muscle weakness or rigidity, initiating an additional compensatory cycle. As a result, compensatory adaptations can become iterative, leading to multiple compensatory cycles that require several levels of postural analysis and adjustments to be fully resolved. Figure S2B schematically illustrates the iterative levels of compensatory cycles presented in Figure S2A, overlaid with the CPM scheme for analysis. This analysis involves identifying the weak and rigid sides (resulting from muscle weakness and rigidity), the loaded side (resulting from load imbalance), and the distressed side (dependent on uneven limb involvement). The red arrow now has a 3D representation, providing a clearer depiction of the iterative compensations described in Figure S2A with inner causes represented at the bottom.

The CPM methodology – postural assessment phase

The CPM postural assessment phase (see Figure S3) should be thought of as a backwards iterative process with the aim of investigating:

1. the causes of muscle rigidity related to the weak side and their consequences on the kinetic chain, considering both overload zones and compensatory postural arrangements;
2. identifying body positions lacking the static postural prerequisites (for example a sufficient ROM reached passively and maintained) that are functional for addressing the specific dynamic needs of the subject examined.

Figure S3. Analysis phase

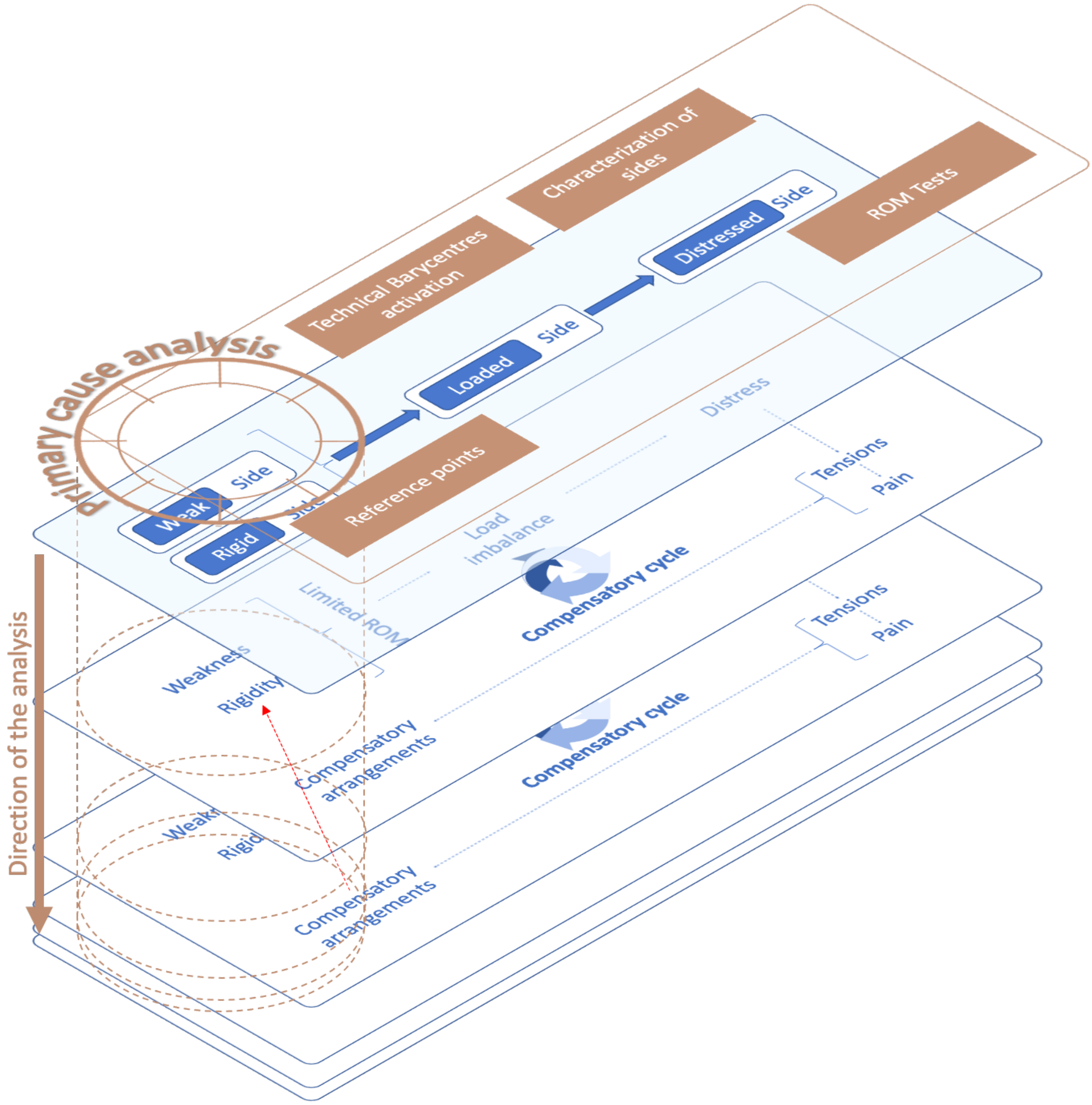

Legend: Figure S3 superimposes a postural analysis scheme onto the compensatory cycle represented in Figure S2. It is important to note that the analysis proceeds in the opposite direction of the compensatory arrangements. Specifically, the most recent compensation is assessed first, with the limited range of motion being the initial focus. The analysis cycle then proceeds counterclockwise and from top to bottom to evaluate and disentangle the superimposed iterative compensatory cycles. The *direction of the analysis* reflects the operator's overall effort and should be viewed as integrated with the *postural reprogramming phase*, which, by removing weaknesses and rigidities, allows for the discovery of primary (deeper) causes.

The key elements of the CPM assessment include:

A) analysis of reference points;

B) analysis of ROM of the joints;

C) characterization of the body sides;

D) analysis of the activation of technical barycentres.

A. The reference points are specific anatomical landmarks that help in identifying postural compensatory arrangements (see Table S1).

Table S1. Evaluable compensatory postural arrangements through reference points

#	Reference plane	Body Position	Endpoint
1, 2	Frontal	Supine [1] and Standing [2] (anterior)	Difference between the two sides of the elevation of the ribcage and the pelvis
3, 4	Sagittal	Supine [3] and Standing [4] (anterior)	Difference between left and right side of the ribcage exposure/advancement
5, 6	Frontal	Prone [5] and Standing [6] (posterior)	Difference between left and right scapula elevation
7, 8	Sagittal	Prone [7] and Standing [8] (posterior)	Difference between left and right scapula exposure/advancement
9	Frontal	Standing	Difference between left and right shoulder elevation
10	Sagittal (or Transverse)	Standing	Difference between left and right shoulder exposure/advancement
11	Transverse	Standing (anterior)	Difference between left and right side of the pelvis exposure/advancement
12, 13	Transverse	Standing (anterior)	Intra- or extra-rotation of the knee – left [12] and right [13]
14, 15	Frontal	Standing (anterior)	Frontal movement (pronation/supination) of ankles – left [14] and right [15]

The CPM methodology does not consider the analysis of reference points as an objective and absolute concept (such as in the case, for example, of an X-ray examination) but frames this assessment within the specific dynamic needs of the examined subject. Their detection occurs through relative measurements that, in various body positions, allow an understanding of whether there is the maintenance or shifting of postural compensatory arrangements.

B. The analysis of joint range of motion (ROM) is conducted through a battery of mobility tests (see Table S2), always in conjunction with the presence of a signal (tension and pain). These signals, in conjunction with the framework provided by reference points, indicated a potential limited availability in another area of the chain.

Table S2. Joint mobility tests

#	Test description	Target	Indicators*,**
1	Forward trunk flexion on ankle plantar flexion (sitting on a box about 30 cm high)	Anterior chain retraction	Ankle and hip ROM
2	Forward trunk flexion on dorsiflexion of pronated foot (sitting on a box about 30 cm high)	Posterior chain retraction	Ankle and hip ROM
3, 4	Forward trunk flexion on dorsiflexion of pronated ankle in extra [3] and intra [4] rotation (sitting on a box about 30 cm high)	Posterior chain retraction due to the innermost [3] and/or outermost [4] rigidity of the thigh musculature or sural triceps	Ankle and hip ROM
5, 6	Flexion and extension of the right [5] and left [6] knee with the sole of the foot constrained with both hands (sitting)	Posterior chain retraction during the step	Knee joint ROM
7, 8	Right [7] and left [8] hip extension with retracted ribcage (ipsilateral knee resting on the ground and flexed to 90 degrees and contralateral limb flexed at the hip and at the knee both to 90 degrees)	Actual hip retraction without the influence of other muscle components of the posterior chain	Hip ROM
9, 10	Flex and extra rotate the gleno-humeral right [9] and left [10] joint with elevated scapula (sitting on a box about 30 cm high)	Right [9] and left [10] shoulder resistance to be related to a global shortening of the chain	Shoulder ROM
11, 12	Passive elevation of the right [11] and left [12] limb (assuming the seated hurdler position*** with knees together and left (right) extended hip)	Evidence of muscles working differently when in crossed chain (as in the step) than when solicited in single-joint exercises	Hip ROM
13	Forward trunk flexion with legs spread apart (sitting on a box about 30 cm high)	Medial chain (adductors) retraction	Hip ROM
14	Forward trunk flexion with legs crossed (sitting on a box about 30 cm high)	Lateral chain (abductors) retraction	Hip ROM

* In addition to the reported indicators, the presence of excessive muscle and tendon tension in the elongating chain and / or annoying pain also elsewhere is recorded for all tests.

** The range of motion is qualitatively assessed when excessive tension or pain occurs.

*** Sitting on the buttocks, left (right) leg bent back, right (left) extended diagonally forward.

C. The characterization of the body sides is conducted using a subset of the reference points and joint mobility tests described earlier (see Table S3).

Table S3. Characterization of the body sides

tableBody side	Identification
Weak side	The side where the ribcage is more exposed in the supine position (**Reference point #3**); if there is no difference, the side in which the ribcage is more elevated with respect to the pelvis in the supine position **(Reference point #1**). In case of an equal involvement of the sides, there is no emerging weak side.
Rigid side	The side where the **joint mobility tests from #1 to #10** show the greater number of muscle rigidities. In case of an equal involvement of the sides, there is no emerging rigid side.
Load side	The side where the greater number of postural compensatory arrangements are shown through **reference points #5–11***. In case of an equal involvement of the sides, there is no emerging load side.
Distressed side	The side where the subject reports pain. In case the subject does not declare any pain or comparable pain in both sides, then the distressed side corresponds to the rigid side.

* With regard to the compensatory postural arrangements #10 (transverse plane) and #11, a rotation to the right (left), is attributed to the right (left) side.

D. The analysis of the activation of technical barycentres.

The battery of tests presented in Table S4 aims to verify whether the technical barycentres activate (i.e. if the subjects perceive that these muscles are contracting) in the two relevant body positions (90° and 0° degrees) in different decubitus positions. Possible causes of incorrect activation, as reported in Feature 5, are dominances in the acting chain, rigidities in the opposed chain or a weakness of the technical barycentres. These tests can lead to four outcomes:

- if the subject reports pain in any area of the body, the test must be repeated in a more comfortable manner (e.g. reducing the joint angles, lowering the opposing force applied by the operator, or modulating exercise conditions to decrease the impact of gravity on body segments);
- if the subject perceives muscular tension in the opposite chain, this result is compared with the assessment from the joint mobility tests to discern whether the tension originates from muscular rigidity or a weak technical barycentre;

- if muscular dominance is identified in the acting chain, the test is repeated after inhibiting the dominant muscle (e.g. by pressing the interested muscle with the edge of the hand in order to avoid the contraction of such muscle, initially applying a force opposite to that which activates the technical barycentre) to understand the actual capacity of the technical barycentre to activate;
- if the examined technical barycentre activates without the subject perceiving any of the aforementioned negative signals, this indicates proper functionality.

Table S4. Analysis of the technical barycentres' activation

#§	Body Position	Joint angle modulation	Opposing force modulation	Technical Barycentre*	Dominance	Resistance
1A	Sitting with trunk at 90° to the floor and upper limbs to the side of the body, with ankles in either plantar- or dorsi-flexed position 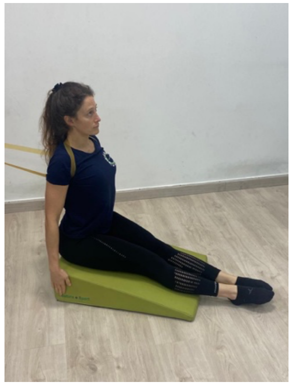 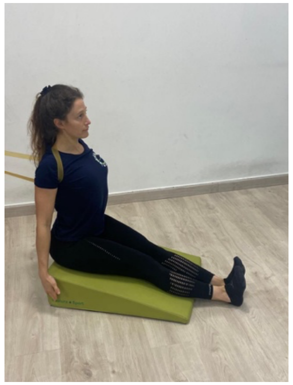	Hip flexion decreased by using a wedge-shaped pillow	Elastic band applied to the trunk (at armpit height), and pulled toward hip extension	Abdominal muscles	Anterior chain of lower body	Posterior chain of lower body

1B	Sitting with trunk at 90° to the floor, shoulder flexed about 180°, and elbow fully extended, with ankles in plantar- or dorsi-flexed position<br>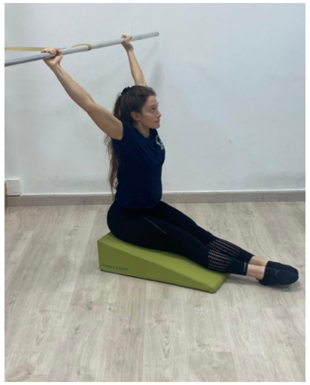 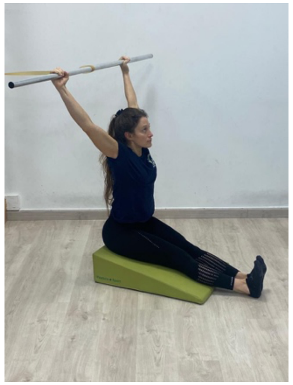	Hip flection decreased by using a wedge-shaped pillow	Elastic band applied to the trunk (at armpit height), or along upper limbs up to the hands, and pulled in the opposite direction of hip anteversion	Abdominal muscles	Anterior chain of lower body	Posterior chain of lower body and upper body anterior chain
2A	Supine with lower limbs at 90° to the floor, upper limbs to the side of the body, with ankles in plantar- or dorsi-flexed position<br>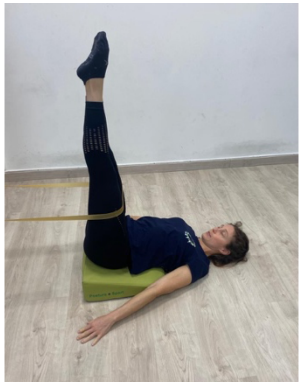 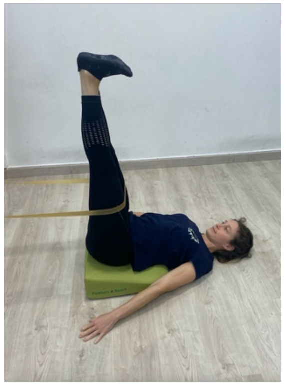	Hip flection decreased by using a wedge-shaped pillow	Elastic band, applied on the lower limbs – from thigh to ankles – pulled toward a hip extension	Abdominal muscles	Anterior chain of lower body	Posterior chain of lower body

2B	Supine with lower limbs at 90° to the floor, shoulder flexed about 180° and elbow fully extended, with ankles in plantar- or dorsi-flexed position 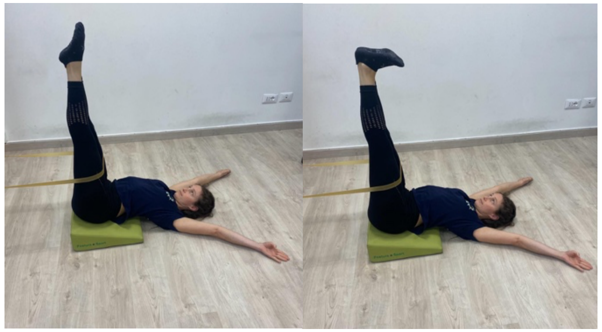	Hip flection decreased by using a wedge-shaped pillow	Elastic band, applied on the lower limbs – from thigh to ankles – pulled toward a hip extension	Abdominal muscles	Anterior chain of lower body	Posterior chain of lower body and upper body anterior chain
3	Sitting with trunk at 90° to the floor, shoulder flexed about 180° and elbow fully extended, with ankles in plantar- or dorsi-flexed position 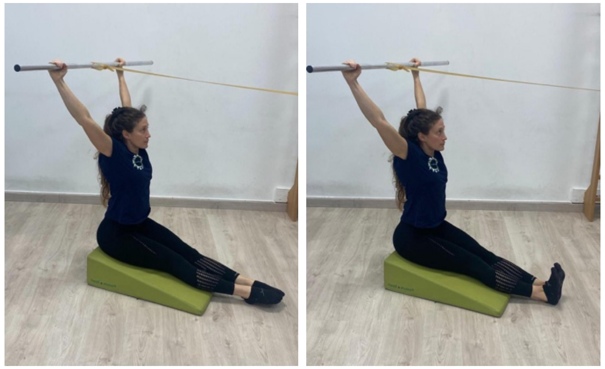	Hip flection decreased by using a wedge-shaped pillow	Elastic band, applied along upper limbs up to the hands, pulled toward a hip flection	Scapular stabilizer muscles with an indirect action** of the abdominal muscles	Anterior chain of lower body	Posterior chain of lower body and upper body anterior chain

4A	In a prone position with hip extended and pelvic retroversion, upper limbs to the side of the body, and one leg raised at a time 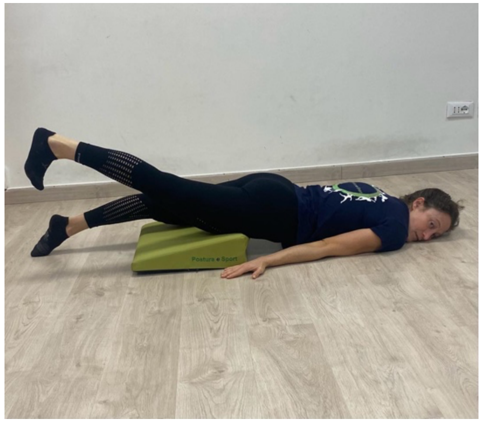	Hip extension decreased by using a wedge-shaped pillow (no more than 20 cm so as not to load the neck muscles)	Use the wedge cushion to shorten the levers by placing it down towards the knees	Hamstring muscles, and abdominal muscles (indirect action§) for pelvis stabilization	Anterior chain of lower body	Anterior chain of lower body and/or posterior chain of lower body (adductors and/or proximal hamstrings)
4B	In a prone position with hip extended and pelvic retroversion, with shoulder flexed about 180° and elbow fully extended, and one leg raised at a time 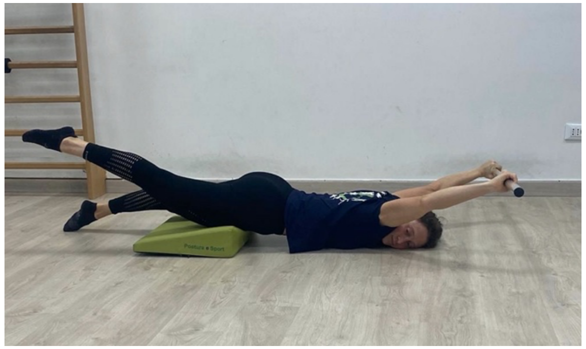	Hip extension decreased by using a wedge-shaped pillow	Use the wedge cushion to shorten the levers by placing it down towards the knees.	Hamstring muscles, abdominal muscles for pelvis stabilization (indirect action§), and shoulder stabilizers	Anterior chain of lower and/or upper body	Anterior chain of lower body and/or posterior chain of lower body (adductors and/or proximal hamstrings)

5A	In a prone position with lower limbs over a 30–50 cm diameter (depending on the anthropometric characteristics of the subject) directional (in the sagittal plane) cylindrical elastic ball, maximum hip extension with pelvic retroversion and arms at 90° to the floor supporting the lifted trunk kept parallel to the floor (knees extended) 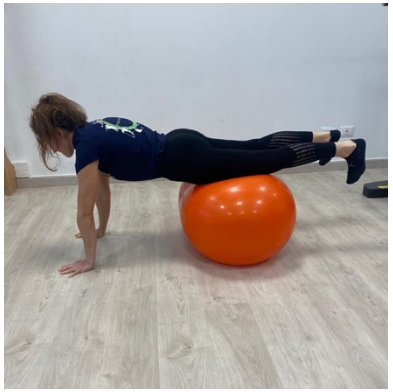	Not applicable	Lower limbs in contact with the ball from thighs to ankles	Abdominal muscles for pelvis stabilization, and hamstring muscles for hip extension	Anterior chain of lower body	Anterior chain of lower body and/or posterior chain of lower body (adductors and/or proximal hamstrings)

5B	Same as 5A but flexing one knee at a time† 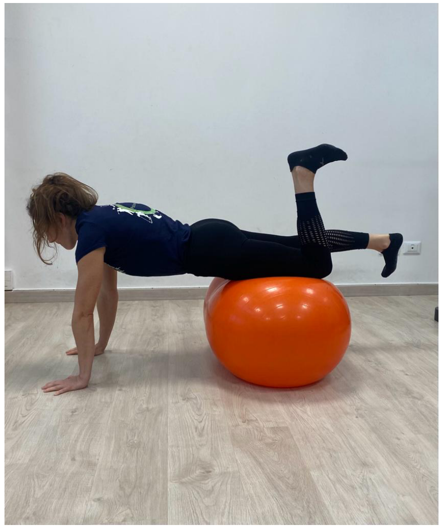	Not applicable	Lower limbs in contact with the ball in the range thighs to knees	Abdominal muscles for pelvis stabilization	Anterior chain of lower body and hamstrings as knee flexors	Anterior chain of lower body

§ Test i-B is executed if i-A is passed successfully.
* Isometric activation unless specified.
** The abdominal muscles are involved in stabilizing the pelvis based on the activation of one or both of the other technical barycentres
† This double role of hamstring muscles in CPM is defined as ***counter-plyometric.***
$ The action of the abdominal muscles is involved in stabilizing the pelvis according to the activation of the other two technical barycentres.

Through the described analyses, the CPM identifies the causes (dominance or resistance with respect to the abdominal muscles or their insufficient activation) of inadequate stabilization of the pelvis and spine in both flexed hip (90°) and extended hip (> 0°) positions. The intervention, without addressing any painful areas, aims to mitigate these causes and, consequently, reduce the need for compensatory postural arrangements. Iteratively, the analysis must be repeated to assess the actual remission of the causes, identifying the emergence of any less impactful, initially not evident, problems (see Figure S3). The analysis phase concludes when the subject is able to stabilize the pelvis and the spine, achieving the ROM of the joints functional to their dynamic needs. Obviously, routine assessment is necessary to verify the maintenance of the achieved conditions.

Among the key elements of the CPM assessment, only the analysis of reference points (A) is operator-dependent. In contrast, the analysis of the ROM of the joints (B) and the analysis of the activation of technical barycentres (D) are based on feedback from the subject. Additionally, the characterization of the body sides (C) combines elements of both A and B. Preliminary data (available on request) on the application of the reference point analysis were collected from a convenience sample of 15 adults with low-back pain, without disabling physical conditions or diagnoses of disc herniation, lumbar stenosis, spinal deformity, fracture, or spondylolisthesis. The assessment was conducted by 30 CPM-certified professionals, allowing for the evaluation of inter-rater reliability and internal consistency. Agreement among different observers assessing the same subjects was measured using intra-class correlation (ICC) with a two-way, single-measure analysis, yielding an ICC score of 0.96, which indicates excellent reliability.

Secondly, recognizing that CPM assessment relies on a combination of multiple reference points, the internal consistency and construct validity of the items was investigated. Factor analysis was used to reveal latent relationships among the reference points and to identify the most representative ones. McDonald's coefficient was employed to estimate internal consistency, with a value exceeding 0.90, indicating high reliability of the identified factors.

The CPM methodology – postural reprogramming phase

As a general approach, CPM postural reprogramming phase proposes personalized intervention strategies to restore the flexion–extension muscle relationships in the acting and / or opposed chains. This is achieved by working on resistant muscles[1] or insufficiently activated technical barycentres (see Feature 5), which were identified in the postural assessment phase. The CPM intervention strategies involve supervised, self-performed gymnastics exercises without the manual interventions typical of physiotherapy approaches. Manual interventions are only employed to assist the subject in maintaining correct positioning while respecting the joint angles required by the exercises. In the postural reprogramming phase, each intervention programme is structured according to a corresponding iteration cycle of the postural assessment phase (customization).

The application of the intervention programme is tailored based on the subject's response to each proposed exercise (individualization).

The supervised exercises are planned and proposed by certified CPM operators based on the following criteria and methods:

---

[1] A resistant muscle is one that is excessively contracted or shortened and resists when an attempt is made to stretch it.

- CUSTOMIZATION
    a. Reduction of neuromuscular tensions in resistant and non-cooperating muscles through the short-term application of a selection of the joint mobility tests, now adopted as stretching exercises.
    b. Restoration, in all the body and decubitus positions described above, of the relationships between the correct neuromuscular tensions and the activation of the abdominal muscles in their role of spine stabilizer; this is achieved through the application of the tests used for the analysis of the technical barycentres' activation, now adopted as strengthening exercises. The exercise load is progressively increased over time in terms of volume and intensity.
    c. Initially, each strengthening exercise is proposed isometrically; once the subject achieves sufficient strength, the same exercises are proposed isometrically but after requiring an eccentric contraction to reach the target position. In the final level, the exercises are performed dynamically.
    d. After the subject successfully activates the abdominal muscles in various body and decubitus positions, both under one direction of load (see Table S4, Exercise 1B) and its opposite (Table S4, Exercise 3), the dynamic capacities of the subject can be further reinforced (see Feature 3). This is achieved through the simultaneous application of back-and-forth rubber bands (superimposed exercises 1B and 3 in Table S4).
- INDIVIDUALIZATION

Annoying pains, excessive tensions, muscular dominances, non-functional asymmetries that may arise during the execution of the exercises must always be investigated and

removed. This should be done using small tools or devices, such as wedge-shaped pillows (for more suitable joint working angles), light hand pressures by the operator directly on a dominant muscle to inhibit it, and small soft pads placed under the lumbar area to promote a comfortable pelvis anteversion in supine decubitus. The use of these supportive strategies should be gradually reduced as postural compensatory arrangements are removed, and the proposed exercises are executed without pains, tensions, dominances and non-functional asymmetries.

Once an acceptable postural balance, functional to the dynamic needs of the examined subject, has been reached, the correct flexion–extension muscle relationships should almost be maintained.

3. References

18. Canali, V. Posture e Sport; Calzetti & Mariucci: Ferriera, Italy, 2014. 25.
44. Carini, F., M., F., P.S., M.M., D.P., T.G. Posture and Posturology, Anatomical and Physiological Profiles: Overview and Current State of Art. - PubMed - NCBI. *Acta Biomed.* **2017**, *88*, 11–16.
45. Kauffman, T.L. Posture. In *Geriatric Rehabilitation Manual*; Churchill Livingstone: London, UK, **2007**; pp. 99–105. https://doi.org/10.1016/B978-0-443-10233-2.50022-5.
46. Massion, J. Postural Control System. *Curr Opin Neurobiol* **1994**, *4*, 877–887, doi:10.1016/0959-4388(94)90137-6.
47. Gaskell, L. Musculoskeletal Assessment. In *Tidy's Physiotherapy*, 5th ed.; Churchill Livingstone: London, UK, **2013**; pp. 207–251. https://doi.org/10.1016/B978-0-7020-4344-4.00011-0.48.
48. Selinger, A. Posture. *Physical Rehabilitation: Evidence-Based Examination, Evaluation, and Intervention*; Elsevier: Amsterdam, The Netherlands, **2007**; pp. 40–63, doi:10.1016/B978-072160361-2.50007-7.
49. Powers, C.M. The Influence of Abnormal Hip Mechanics on Knee Injury: A Biomechanical Perspective. *Journal of Orthopaedic and Sports Physical Therapy* **2010**, *40*, 42–51, doi:10.2519/jospt.2010.3337.
50. Kratenová, J.; Zejglicová, K.; Malý, M.; Filipová, V. Prevalence and Risk Factors of Poor Posture in School Children in the Czech Republic. *J Sch Health* **2007**, *77*, 131–137, doi:10.1111/j.1746-1561.2007.00182.x.
51. Lee, J.H. Effects of Forward Head Posture on Static and Dynamic Balance Control. *J Phys Ther Sci* **2016**, *28*, 274–277, doi:10.1589/jpts.28.274.

52. Wirth, B.; Knecht, C.; Humphreys, K. Spine Day 2012: Spinal Pain in Swiss School Children- Epidemiology and Risk Factors. *BMC Pediatr* **2013**, *13*, 159. doi:10.1186/1471-2431-13-159.
53. Brumagne, S.; Janssens, L.; Janssens, E.; Goddyn, L. Altered Postural Control in Anticipation of Postural Instability in Persons with Recurrent Low Back Pain. *Gait Posture* **2008**, *28*, 657–662, doi:10.1016/j.gaitpost.2008.04.015.
54. Watson, A.W. Sports Injuries Related to Flexibility, Posture, Acceleration, Clinical Defects, and Previous Injury, in High-Level Players of Body Contact Sports. *Int J Sports Med* **2001**, *22*, 222–225, doi:10.1055/s-2001-16383.
55. Wang, H.; Gao, X.; Shi, Y.; Wu, D.; Li, C.; Wang, W. Effects of Trunk Posture on Cardiovascular and Autonomic Nervous Systems: A Pilot Study. *Front Physiol* **2022**, *13*, 1009806. doi:10.3389/FPHYS.2022.1009806.
56. Zafar, H.; Albarrati, A.; Alghadir, A.H.; Iqbal, Z.A. Effect of Different Head-Neck Postures on the Respiratory Function in Healthy Males. *Biomed Res Int* **2018**, *2018*, 4518269. doi:10.1155/2018/4518269.
57. Lin, F.; Parthasarathy, S.; Taylor, S.J.; Pucci, D.; Hendrix, R.W.; Makhsous, M. Effect of Different Sitting Postures on Lung Capacity, Expiratory Flow, and Lumbar Lordosis. *Arch Phys Med Rehabil* **2006**, *87*, 504–509, doi:10.1016/J.APMR.2005.11.031.
58. Łastowiecka-Moras, E. How Posture Influences Venous Blood Flow in the Lower Limbs: Results of a Study Using Photoplethysmography. *Int J Occup Saf Ergon* **2017**, *23*, 147–151, doi:10.1080/10803548.2016.1256938.
59. Dainese, R.; Serra, J.; Azpiroz, F.; Malagelada, J.R. Influence of Body Posture on Intestinal Transit of Gas. *Gut* **2003**, *52*, 971–974, doi:10.1136/GUT.52.7.971.
60. Nair, S.; Sagar, M.; Sollers, J.; Consedine, N.; Broadbent, E. Do Slumped and Upright Postures Affect Stress Responses? A Randomized Trial. *Health Psychol* **2015**, *34*, 632–641, doi:10.1037/HEA0000146.
61. Hirata, K.; Yamadera, R.; Akagi, R. Associations between Range of Motion and Tissue Stiffness in Young and Older People. *Med Sci Sports Exerc* **2020**, *52*, 2179–2188, doi:10.1249/MSS.0000000000002360.
62. Unione Nazionale Chinesiologi Chi Siamo. Available online: https://www.unc.it/noi-unione-nazionale-chinesiologi (accessed on 4 February 2022).
63. Ruivo, R.M.; Carita, A.I.; Pezarat-Correia, P. The Effects of Training and Detraining after an 8 Month Resistance and Stretching Training Program on Forward Head and Protracted Shoulder Postures in Adolescents: Randomised Controlled Study. *Man Ther* **2016**, *21*, 76–82, doi:10.1016/J.MATH.2015.05.001.
64. Kim, D.; Cho, M.; Park, Y.; Yang, Y. Effect of an Exercise Program for Posture Correction on Musculoskeletal Pain. *J Phys Ther Sci* **2015**, *27*, 1791–1794, doi:10.1589/JPTS.27.1791.
65. Clippinger-Robertson, K. Kinesiology and Injury Prevention: Every Dancer's Guide. *J Phys Educ Recreat Dance* **1986**, *57*, 50–53, doi:10.1080/07303084.1986.10606133.
66. American Kinesiology Association Available online: https://americankinesiology.org/about-us/ (accessed on 3 March 2024).
67. Oba, K.; Ohta, M.; Mani, H.; Suzuki, T.; Ogasawara, K.; Samukawa, M. The Effects of Static Stretching On Dynamic Postural Control During Maximum

Forward Leaning Task. *J Mot Behav* **2023**, *55*, 594–602, doi:10.1080/00222895.2021.1909529.
68. Steindler, A. *Kinesiology of the Human Body under Normal and Pathological Conditions*; Charles C Thomas: Springfield, Illinois, 1955;
69. Mier, A.; Brophy, C.; Estenne, M.; Moxham, J.; Green, M.; De Troyer, A. Action of Abdominal Muscles on Rib Cage in Humans. *J Appl Physiol (1985)* **1985**, *58*, 1438–1443, doi:10.1152/jappl.1985.58.5.1438.
